# Induction of Immune Surveillance of the Dysmorphogenic Lens

**DOI:** 10.1038/s41598-017-16456-5

**Published:** 2017-11-24

**Authors:** Caitlin M. Logan, Caitlin J. Bowen, A. Sue Menko

**Affiliations:** Thomas Jefferson University, Department of Pathology, Anatomy and Cell Biology, Philadelphia, Pennsylvania 19107 United States

## Abstract

The lens has been considered to be an immune privileged site not susceptible to the immune processes normally associated with tissue injury and wound repair. However, as greater insight into the immune surveillance process is gained, we have reevaluated the concept of immune privilege. Our studies using an N-cadherin lens-specific conditional knockout mouse, N-cad^Δlens^, show that loss of this cell-cell junctional protein leads to lens degeneration, necrosis and fibrotic change, postnatally. The degeneration of this tissue induces an immune response resulting in immune cells populating the lens that contribute to the development of fibrosis. Additionally, we demonstrate that the lens is connected to the lymphatic system, with LYVE(+) labeling reaching the lens along the suspensory ligaments that connect the lens to the ciliary body, providing a potential mechanism for the immune circulation. Importantly, we observe that degeneration of the lens activates an immune response throughout the eye, including cornea, vitreous humor, and retina, suggesting a coordinated protective response in the visual system to defects of a component tissue. These studies demonstrate that lens degeneration induces an immune response that can contribute to the fibrosis that often accompanies lens dysgenesis, a consideration for understanding organ system response to injury.

## Introduction

N-cadherin has been extensively studied for its role in development^1–3^, tissue morphogenesis^2,4,5^ and cancer progression^6,7^. It, along with other cell-cell adhesion junctions, provide the cellular interaction that is necessary to create and maintain structural integrity of a tissue^8,9^. Our studies of the lens conditional N-cadherin knockout (N-cad^Δlens^) show that N-cadherin is necessary for proper lens development^10^ with its loss leading to aberrant fiber cell elongation and dysmorphogenesis that eventually results in cell disorganization and death. Since in this conditional knockout N-cadherin is lost only in the lens, a tissue centrally located in the eye, the N-cad^Δlens^ mouse provided the unique opportunity to investigate the visual system’s response to the increasing dysmorphogenesis of one of its component parts.

The responses to tissue pathogenesis or injury include critical homeostatic processes that underlie tissue repair and regeneration. In most tissues, response to the pathogenic disruption of normal tissue architecture comes from both innate and adaptive immune systems, including the recruitment of immune cells^11–13^. However, in tissues that have been classified as “immune privileged”, including the lens and other tissues of the eye^11,14,15^, the potential impact of immune surveillance in response to degeneration of these tissues is not often considered. Recently, the notion that tissues have immune privilege has been challenged, with studies suggesting that the brain and the eye may in fact be subject to immune surveillance and lymphatic drainage, and instead of immune privilege possess mechanisms promoting immunoquiescence^16–19^. In the cornea, like the lens, the absence of a vasculature is essential to its transparency. Immune privilege of the cornea includes a tolerance to foreign antigens through a complex process referred to as anterior chamber-associated immune deviation^20^. Yet, there of sources of immune cells that surveille the cornea, including the lymphoid tissues of the eyelids and conjunctiva^21^, with high numbers of immune cells being present in the tears that contact the cornea surface^22^. In addition, in response to injury, innate immune cells that reside in the peripheral cornea rapidly populate the central cornea^23–25^. Here, we examine the possibility that the lens is also a tissue subject to immune cell surveillance and invasion. Understanding the lens’ potential as a target of immune reaction could give a deeper knowledge of the mechanisms of lens-specific injury response, including fibrotic outcomes in cataract and Posterior Capsule Opacification (PCO), as well as the overall process of immune surveillance and signaling to protect an organ such as the eye from the dysgenesis of one of its component tissues.

## Results

### Embryonic dysmorphogenesis of lens-specific conditional N-cadherin knockout leads to postnatal degeneration and lens opacity

**Figure 1:**
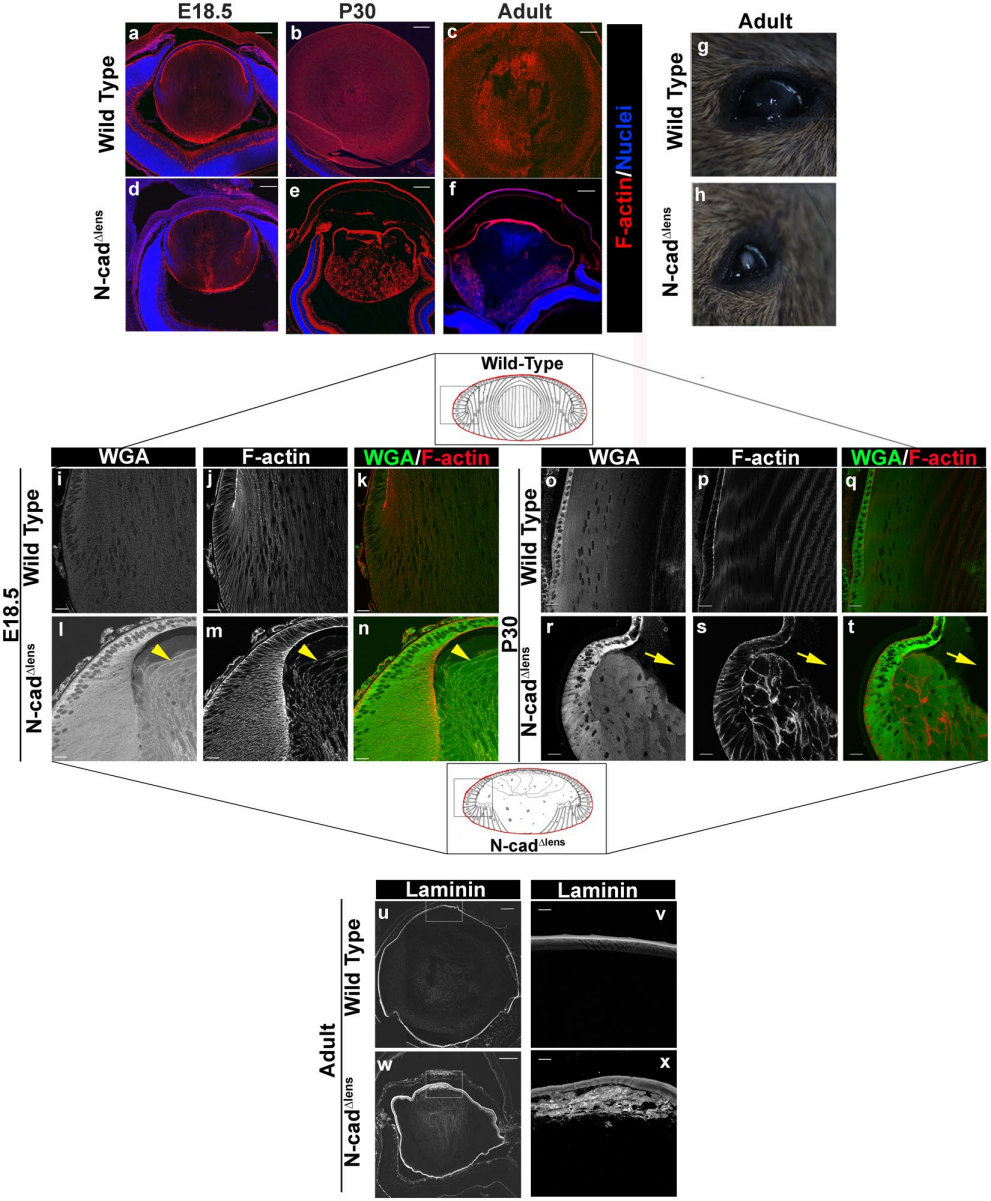
N-cadherin lens-specific conditional knockout results in lens dysmorphogenesis that progresses with time and results in lens opacity. Cryosections of E18.5 (**a**,**d**), P30 (**b**,**e**), or adult (**c**,**f**) wildtype (**a**–**c**) or N-cad^Δlens^ (**d**–**f**) eyes were stained for F-actin (red) and nuclei (blue). F-actin labeling, which highlights lens cytoarchitecture, demonstrated that lenses of N-cad^Δlens^ mice experienced dysmorphogenesis that progressed over time, with a failure of secondary lens fiber cells to elongate beyond the lens equator at E18.5 (**d**), disorganization of secondary lens fiber cells and degradation of primary lens fiber cells at P30 (**e**), and severe breakdown of the central compartment of the lens in the adult (**f**). Photography of the eyes of adult wildtype (**g**) and N-cad^Δlens^ (**h**) mice showed the presence of opacities in the lenses of N-cad^Δlens^ mice. Cryosections of E18.5 (**i**–**n**) and P30 (**o**–**t**) eyes from wildtype (**i**–**k**,**o**–**q**) and N-cad^Δlens^ (**l**–**n**,**r**–**t**) mice were stained for Wheat Germ Agglutinin (WGA) and F-actin. F-actin and WGA highlight the dysmorphogenesis of primary lens fiber cells at E18.5 (**l**–**n**, arrowheads denote disorganized, swollen primary fiber cells). By P30, in lenses of N-cad^Δlens^ eyes, the secondary fiber cells were dysmorphogenic and most cells were no longer attached to the lens capsule, yet these cells remained attached to one another (**r**–**t**). Primary fiber cells had been lost (**r**–**t**, arrows). Cryosections of adult mouse eyes from wildtype (**u**,**v**) and N-cad^Δlens^ (**w**,**x**) mice were immunostained for laminin, a principle component of the lens capsule. Lenses in both wildtype and N-cad^Δlens^ eyes were surrounded by intact laminin-positive capsule. High magnification imaging of laminin labeling of the N-cad^Δlens^ mouse eyes (**x**) showed a thickened lens capsule and aberrant synthesis of laminin by lens epithelial cells. Boxed in regions of the models denote areas represented in the images. (Mag bars in **a**–**f**, **u**,**w** = 200 μm, in **i**–**t**,**v**,x = 20 μm).

The lens-specific N-cadherin conditional knockout (N-cad^Δlens^), in which N-cadherin is lost by E13.5, causes a severe morphogenetic phenotype characterized by a failure of secondary lens fiber cells to elongate due to their inability to migrate along the apical surfaces of the anterior lens epithelium and form an Epithelial Fiber cell Interface (EFI)^10^. This defect results in the progressive loss of tissue structure, in great part due to the disorganization of the first cells to differentiate in the lens, the primary lens fiber cells. By E18.5, the N-cad^Δlens^ lenses begin to exhibit signs of degeneration with the appearance of pyknotic, TUNEL-positive nuclei in primary fiber cells^10^. At this stage, there emerges a dichotomy between the secondary lens fiber cells that exhibit failure of migration and elongation but remain cohesive through lateral interactions and the primary lens fiber cells that lose organizational integrity and their interaction with the anterior epithelium (compare Fig. 1a to d). This phenotype is highlighted when lenses of the E18.5 N-cad^Δlens^ mouse are co-labeled for the lectin WGA, which binds to sialic acid and N-acetylglucosaminyl residues (Fig. 1l,n), and F-actin (Fig. 1m,n), revealing extensive disorganization and swelling of primary fiber cells (Fig. 1l-n, arrowheads).

The dysgenesis of the lens that occurs during development in the N-cad^Δlens^ mouse led to severe dysmorphogenesis of the lens in the postnatal N-cad^Δlens^ mouse (postnatal day (P)30, Fig. 1e) and significant degeneration of lens tissue in the adult (Fig. 1f). At P30, significant degeneration and cell loss had occurred in the region that had been occupied by the primary lens fiber cells (Fig. 1r–t, arrows). The lens epithelium remained intact, albeit with some changes in individual cell shape, and linked to a population of highly disorganized secondary fiber cells that maintained cell-cell cohesion (Fig. 1r–t). While the N-cad^Δlens^ mouse lenses retained a residual architecture, postnatal lens growth was severely blocked, as is evident when these lenses (Fig. 1e,r–t) are compared to wild-type lenses (Fig. 1b,o–q). As these mice continue to age, their lenses exhibited further deterioration, with increased loss of cellular integrity in the center of the lens in the adult, evidenced by a lack of F-actin staining (Fig. 1f). The dysmorphogenesis and degeneration that occurs in adult lenses in the N-cad^Δlens^ mouse resulted in lens opacities (Fig. 1h). Since other lens knockout phenotypes that cause degeneration of this tissue result in disruption of the lens capsule^26–28^, we investigated whether the lens capsule remained intact in lenses of adult N-cad^Δlens^ mice. Immunolabeling for laminin, a major component of the basement membrane capsule, demonstrated the presence of an intact capsule surrounding the lenses of adult N-cad^Δlens^ mice, and a thickening of the anterior lens capsule (compare Fig. 1w,x to u,v). High magnification imaging of N-cad^Δlens^ eyes also revealed the aberrant expression of laminin within the lens that appears to result from the nonpolarized secretion of laminin into the intracellular space by a population of disorganized cells (Fig. 1x).

### Dysgenic Secondary Lens Fiber Cells in the Postnatal N-cad^Δlens^ Mouse Maintain Cohesion and Differentiation-Specific Protein Expression

**Figure 2:**
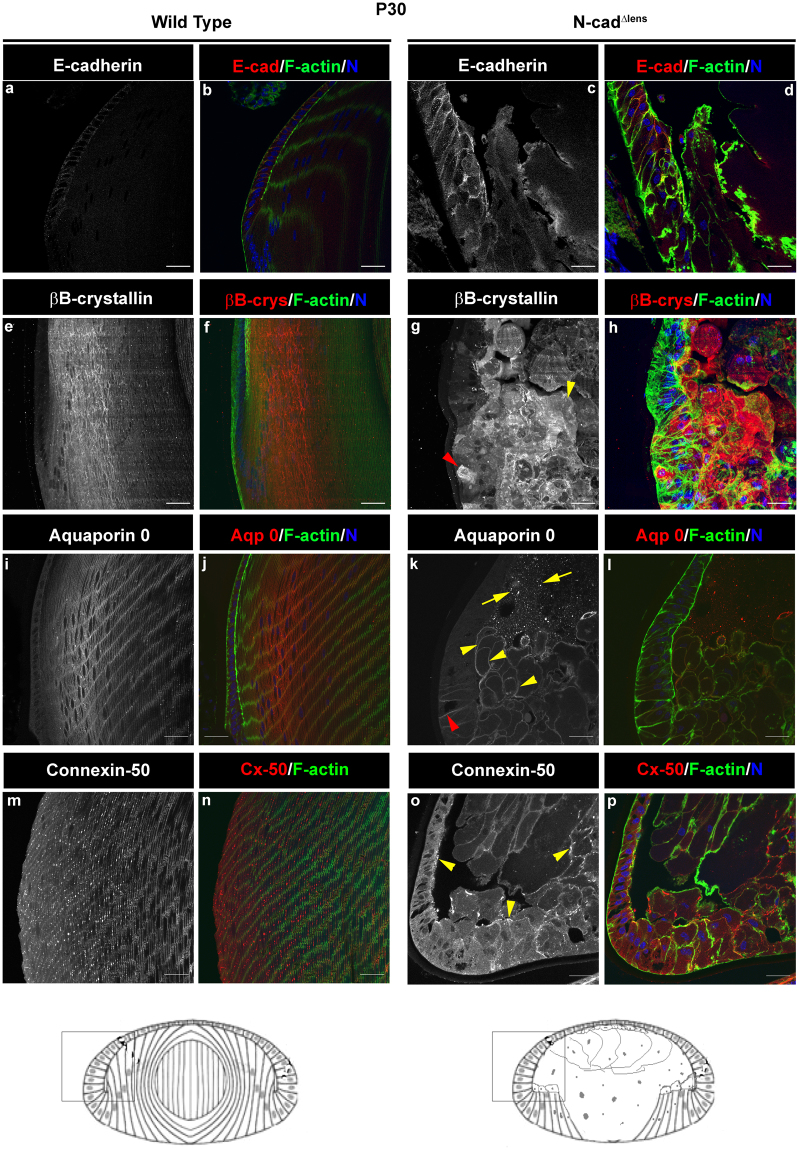
Dysmorphogenic secondary fiber cells of postnatal lenses from N-cad^Δlens^ mice maintain expression of differentiation-state specific molecules. Cryosections of eyes from P30 wildtype (**a,b,e,f,i,j,m,n**) and N-cad^Δlens^ (**c,d,g,h,k,l,o,p**) mice were immunostained for E-cadherin (**a**–**d**), βB-crystallin (**e**–**h**), Aquaporin-0 (**i**–**l**, Aqp0), or Connexin-50 (**m**–**p**, Cx50) and labeled for F-actin (**b,d,f,h,j,l,n,p**) and nuclei (**b,d,f,h,j,l,n,p**). E-cadherin levels were elevated in lenses of N-cad^Δlens^ mice (**c,d**), with evidence of E-cadherin, normally an epithelium-specific protein, extending into lens fiber cells. Expression of βB-crystallin, a fiber cell-specific protein was maintained in the fiber cells of the lenses from N-cad^Δlens^ eyes (**g,h**, yellow arrowhead denotes fiber cells in **g**), showing that these cells express lens differentiation-state specific proteins. Aquaporin-0 also was expressed by lens fiber cells in both wildtype eyes (**i,j**) and N-cad^Δlens^ eyes (**k,l**, yellow arrowhead denotes Aqp0 at lateral fiber cell interfaces in **k**). In the knockout lenses, Aqp0 also had a diffuse and speckled labeling pattern in the central area of the lens that had originally been occupied by primary lens fiber cells (k, arrows). Premature expression of βB-crystallin and Aquaporin-0 was detected in cells of the equatorial epithelium (**g,k**, red arrowheads). Connexin-50 was upregulated in the N-cad^Δlens^ eye lens, with strong expression especially at the apical borders of the lens epithelium and at cell-cell interfaces of lens fiber cells (**o**, arrowheads). Boxed in regions of the models denote areas represented in the images. (mag bars = 20 μm).

Lens development in the N-cad^Δlens^ mouse is compromised because of a failure of differentiating secondary fiber cells to migrate along the EFI and elongate^10^. Despite their short stature, elongating only as far as the lens equator, their apical domains are aligned and they maintain lateral contacts and linearity throughout development. As in the normal embryonic lens, the elongation defective fiber cells in embryonic N-cad^Δlens^ mouse lenses express differentiation-state specific proteins such as βB-crystallin, and the lens channel proteins Aquaporin-0 (Aqp0) and Connexin (Cx)-50, both essential to lens function, still localize along their cell-cell interfaces^10^. Atypical of normal lenses, newly differentiating fiber cells in N-cad^Δlens^ embryonic lenses retain E-cadherin junctions normally found only in cells of the lens epithelium^10^. Since we now show that postnatal N-cad^Δlens^ secondary lens fiber cells exhibit significant dysmorphogenesis by P30, we investigated whether these cells still maintained the differentiation profile they exhibited during development. Sections from both wildtype and N-cad^Δlens^ mouse eyes at P30 were immunolabeled for E-cadherin, βB-crystallin, Aqp0 and Cx50 (Fig. 2). Confocal microscopy imaging of their lenses showed that E-cadherin junctions were maintained at cell-cell borders of cells in the lens epithelium, and were present only at a low level at cell-cell interfaces of the secondary fiber cells just adjacent to the equatorial epithelium (Fig. 2c,d). Despite their dysmorphogenesis, at P30 the N-cad^Δlens^ secondary lens fiber cells maintained expression of the lens differentiation-specific protein βB-crystallin (Fig. 2g,h, yellow arrowhead in g), and high levels of Aqp0 were localized along their cell-cell interfaces (Fig. 2k,l, yellow arrowheads in k). Interestingly, the cells in the region of the adjacent equatorial epithelium from where new secondary fiber cells are sourced also expressed Aqp0 along their lateral borders (Fig. 2k, red arrowhead) and the lens fiber cell protein βB-crystallin (Fig. 2g, red arrowhead). In addition, the morphology of these equatorial epithelial cells was somewhat elongated, which together with their expression of Aqp0 and βB-crystallin suggests that the loss of secondary fiber cell normality in lenses of the postnatal p30 N-cad^Δlens^ mouse results in premature fiber cell formation in the cells of the equatorial epithelium.

These studies also provided insight into the fate of the primary fiber cells that had originally populated the center of the lens. Labeling for WGA and F-actin showed that, by E18.5, the primary fibers located in the center of the lens already were disorganized and swollen (Fig. 1l–n, arrowheads). The greatly diminished labeling for F-actin and WGA from this region of the lens by P30 (Fig. 1r–t, arrows) suggested that most of this cell population had degenerated. Immunolabeling analysis for Aqp0 at P30 revealed a diffuse and speckled staining pattern for this lens protein in the central region of the lens (Fig. 2k, yellow arrows). This finding provides evidence that the region previously occupied by N-cad^Δlens^ primary fiber cells is now filled with proteinaceous material remaining following degeneration of these cells. This outcome also may explain why the anterior epithelial cells of postnatal lenses in the N-cad^Δlens^ mice do not collapse upon the residual, disorganized secondary fiber cell mass following the loss of the primary fiber cells and these lenses retain a semblance of their shape (see Fig. 1e,f).

Given the severity of dysmorphogenesis of the N-cad^Δlens^ secondary fiber cells, it was surprising to find that these cells still maintained cell-cell adhesion. While Aqp0 and Cx50 are best known for their function as channel proteins, they can also have cell adhesive functions^29–31^. Localization of Aqp0 to cell-cell borders of secondary fiber cells at P30 suggests that this channel protein might be responsible for maintaining the cohesion of these dysmorphogenic fiber cells (Fig. 2k, yellow arrowheads). Immunolabeling for Cx50 showed that it too localized to cell-cell borders between neighboring N-cad^Δlens^ fiber cells. In lenses of N-cad^Δlens^ mice, Cx50 was organized as puncta or short linear structures in the secondary fiber cells just adjacent to the epithelium, along the cell-cell interfaces of the dysmorphogenic secondary fiber cells more internal to the lens, as well as along apical surfaces of the lens epithelium (Fig. 2o, all denoted by yellow arrowheads). Interestingly, the knockout of connexins Cx46 and Cx50 results in swelling and degeneration of inner lens fiber cells, while peripheral fiber cells continue to develop normally, even postnatally^32^, a strikingly similar phenotype to the lenses of N-cad^Δlens^ mice. It is possible therefore that these two distinct molecules may have coordinate roles in maintaining lens cytoarchitecture and viability.

### Apoptotic cell death is followed by necrosis in the N-cad^Δlens^ postnatal lens

**Figure 3:**
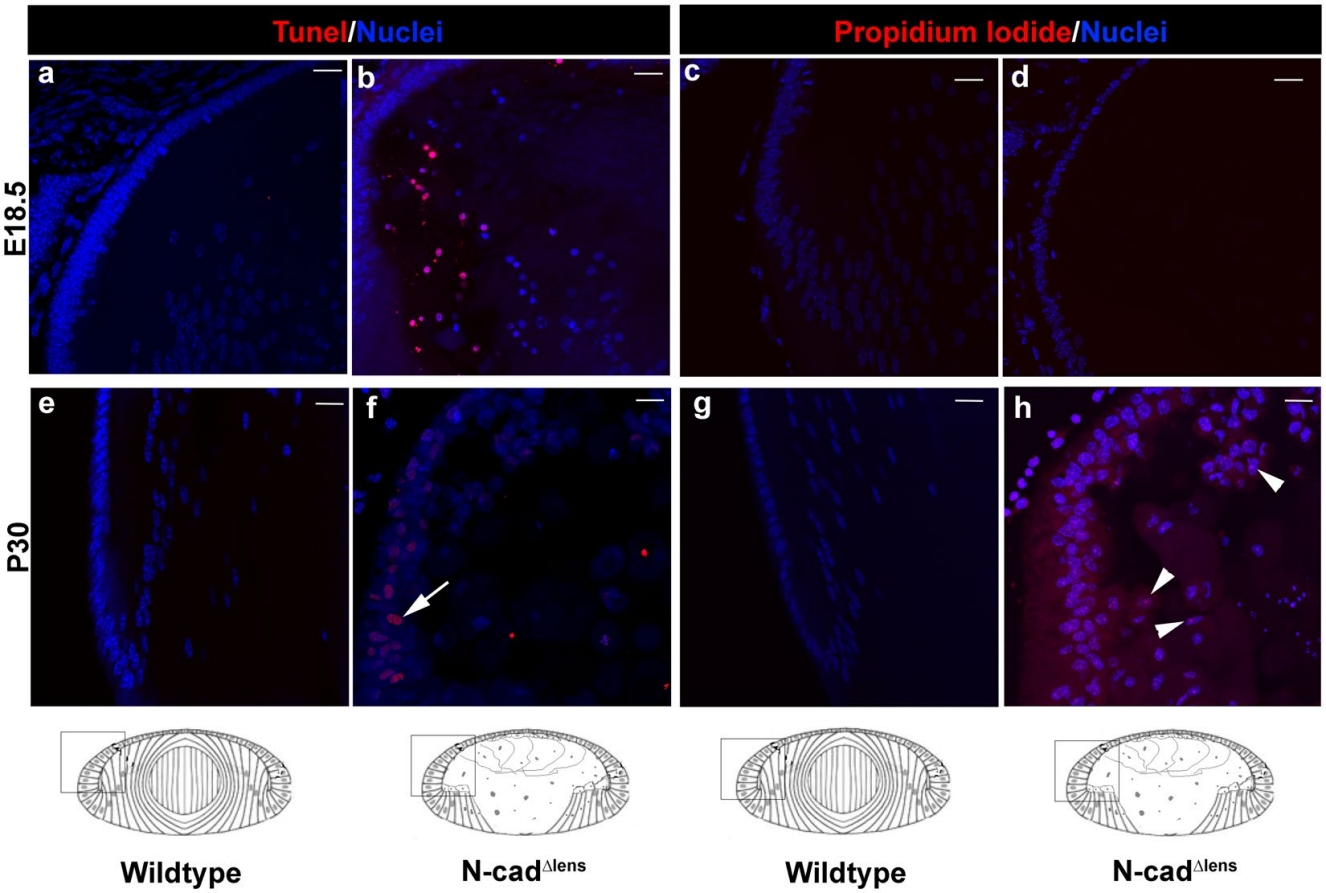
Conditional knockout of N-cadherin in the lens results in lens cell apoptosis followed by necrosis. Cryosections of E18.5 (**a**–**d**) and P30 (**e**–**h**) eyes from wildtype (**a,c,e,g**) and N-cad^Δlens^ (**b,d,f,h**) mice were stained for TUNEL (**a,b,e,f**), propidium iodide (**c,d,g,h**) and nuclei (**a**–**h**). Wildtype lenses showed no evidence of either TUNEL or propidium iodide staining. At E18.5, TUNEL positive labeling was detected in some primary fiber cells of lenses from N-cad^Δlens^ mice (**b**, red), but no labeling for propidium iodide was detected (**d**), showing that the primary fiber cells undergoing apoptosis were not necrotic. By P30 TUNEL labeling was extended to cells in the equatorial epithelium of lenses in N-cad^Δlens^ eyes (**f**, arrow), and these cells, as well as cells in the central part of these lenses, were labeled with propidium iodide (**h**, arrowheads), showing a progression from apoptosis to necrosis in N-cadherin knockout lenses as they age. Boxed in regions of the models denote areas represented in the images. (mag bars = 20 μm).

Our previous studies of the embryonic N-cad^Δlens^ mouse demonstrated that the dysmorphogenesis caused by loss of N-cadherin resulted in apoptosis of a subpopulation of primary lens fiber cells at E18.5, the latest stage of embryonic development^10^. We now investigated whether the progressive degeneration of the lens in the postnatal N-cad^Δlens^ mouse resulted in further cell death and necrosis. Sections from E18.5 and P30 eyes from wildtype and N-cad^Δlens^ mice were labeled by TUNEL assay to assess apoptotic cell death, and with Propidium Iodide (PI) to determine whether the cells had become necrotic, and imaged by confocal microscopy (Fig. 3). At E18.5, when TUNEL-positive cells are first detected in N-cad^Δlens^ primary fiber cells (Fig. 3b), there was no evidence of necrosis (Fig. 3d). By P30, as the lenses continued to develop postnatally, TUNEL-positive cells were now also detected in the equatorial epithelium (Fig. 3f, arrow), and the cells in this region of the lens, along with primary fiber cells in the center of the lens, were found to be positive for PI (Fig. 3h, arrowheads). These results suggest that over time the dysmorphogenesis of lens fiber cells is accompanied by cell death and necrosis associated with the observed degeneration of primary lens fiber cells.

### Degenerative process in the postnatal N-cad^Δlens^ mouse lens leads to fibrosis

**Figure 4:**
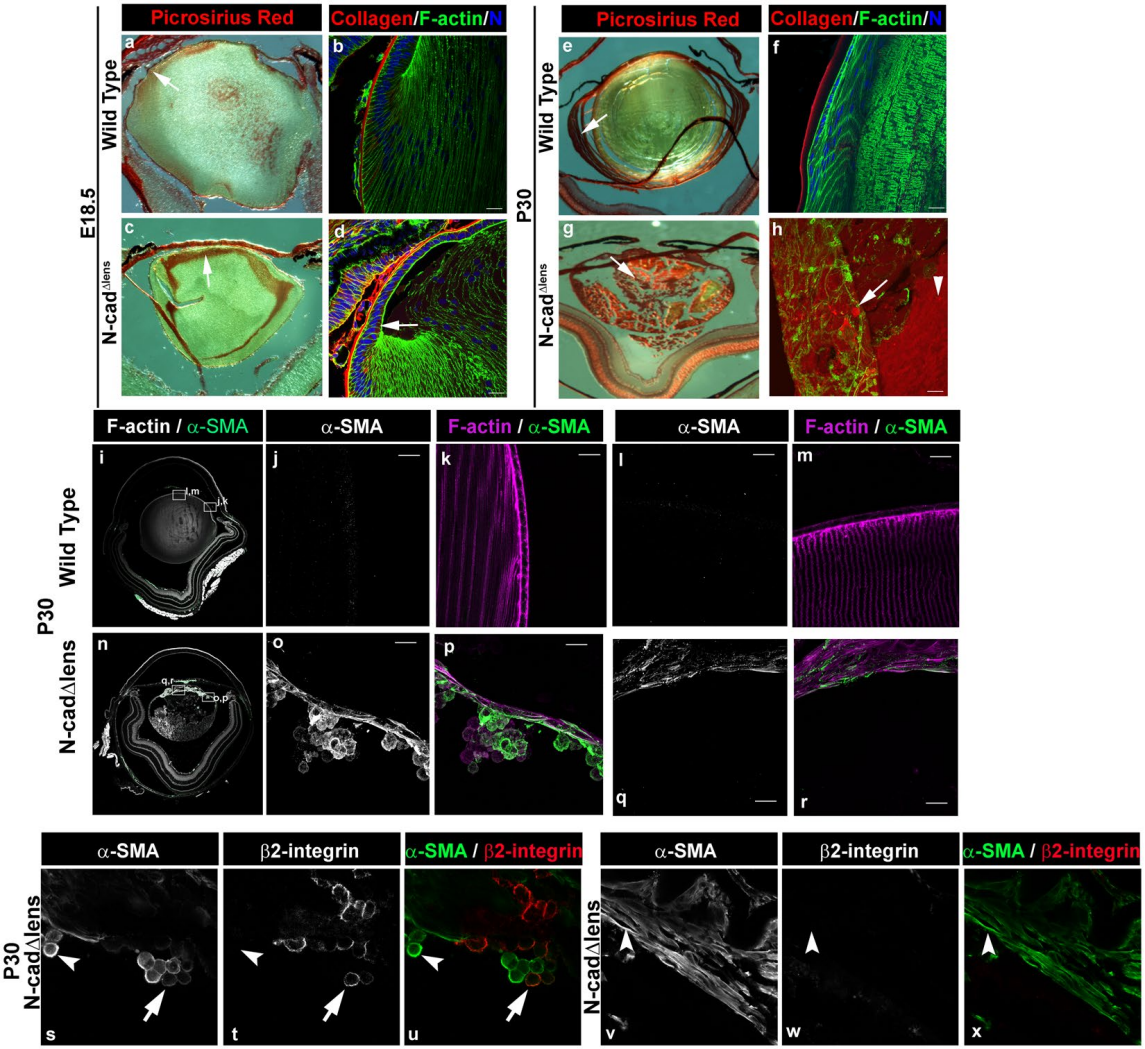
Fibrosis of N-cadherin conditional knockout lenses evidenced by expression of collagen I and presence of α-smooth muscle actin-positive cells. Cryosections of E18.5 (**a**–**d**) and P30 (**e**–**x**) wildtype (**a,b,e,f,i**–**m**) and N-cad^Δlens^ (**c,d,g,h,n**–**x**) eyes were stained for collagen I and III with picrosirius red (**a,c,e,g**), immunolabeled for collagen I and co-labeled for F-actin and nuclei (**b,d,f,h**), or immunolabeled for α-smooth muscle actin (α-SMA) (**i**–**s,u,v,x**) and co-labeled for F-actin (**i,k,m,n,p,r**) or β2 integrin (**t,u,w,x**). Picrosirius red staining showed low-level labeling in the equatorial epithelium of normal lenses at E18.5 (**a**, arrow) and increased collagen labeling in lenses of N-cad^Δlens^ eyes at E18.5, most prominently in the area beneath the anterior lens epithelium (**c**, arrow). Collagen I immunolabeling was detected in lens equatorial epithelial cells of N-cad^Δlens^ eyes at E18.5 (**d**, arrow). At P30, picrosirius red labeling in wildtype lenses was extended to new fiber cells (**e**, arrow) and was present throughout the lens in N-cad^Δlens^ eyes (**g**, arrow). Immunolabeling of P30 N-cad^Δlens^ eyes revealed that collagen I was expressed by cells of the equatorial epithelium (**h**, arrow) and the remaining cortical fiber cells (**h**, arrowhead). No wildtype lenses showed any evidence of α-SMA labeling at P30 (**i**–**m**, panels in **j,k**,**l,m** are from boxed regions in **i**); however, there were many α-SMA+ cells in the lenses of N-cad^Δlens^ eyes, primarily located to the region just beneath the equatorial epithelium (n–p, panels in o,p are from boxed region of n) and the region beneath the anterior epithelium (n,q,r, panels in q,r are from boxed region of n), including cells with prominent α-SMA+ stress fibers (**q,r**). P30 N-cad^Δlens^ eyes were co-immunolabeled for the immune cell adhesion receptor β2 integrin (CD18, **t,u,w,x**) and α-SMA (**s,u,v,x**). While α-SMA positive rounded cells seen beneath the equatorial epithelium were often also positive for β2-integrin (**s**–**u**, arrows), a subset of rounded cells that expressed high levels of α-SMA were negative for β2-integrin (**s**–**u**, arrowheads). α-SMA positive cells with a mesenchymal morphology did not express β2-integrin (**v**–**x**, arrowhead). (mag bars = 20 μm).

Lens degeneration and the formation of lens opacities are often associated with molecular changes associated with fibrosis. Similarly, lens injury resulting from cataract surgery elicits a wound-healing response that frequently results in the fibrotic condition known as PCO. As in other tissues, the development of fibrosis in the lens typically is associated with the production of collagen I, which changes the tissue milieu, and the emergence of α-smooth muscle actin (α-SMA) positive myofibroblasts^33–35^. We investigated whether the dysgenesis of lenses that occurs in response to development in the absence of N-cadherin creates a fibrotic-inducing environment. Wildtype and N-cad^Δlens^ eyes from E18.5 and P30 mice were sectioned and stained for picrosirius red, a dye that associates along cationic collagen fibers and identifies the presence of fibrillar collagens I and III^36,37^. At E18.5 there was already an aberrant accumulation of collagen in regions internal to lenses of N-cad^Δlens^ eyes, most predominantly in an area just beneath the lens epithelium (Fig. 4c, arrow), which corresponded to a region of primary fiber cell dysgenesis and apoptotic cell death (see Figs. 1n and 3b). Interestingly, there is low-level picrosirius red staining of the equatorial epithelium in E18.5 wildtype lenses (Fig. 4a, arrow), which supports earlier reports of collagen production by these cells^38^. Immunolabeling of E18.5 N-cad^Δlens^ eyes with antibody to collagen I (Fig. 4d) showed an increased level of collagen I in the lens capsule, and increased expression of collagen I by cells in the equatorial epithelium (Fig. 4d, arrow) as well as by a subset of newly differentiating cells in the transition zone. Outside of the lens capsule, there was little labeling for collagen I with this antibody in E18.5 wild-type lenses (Fig. 4b). The localization of collagen I to the anterior aspects of cells of the equatorial epithelium of lenses in E18.5 N-cad^Δlens^ eyes suggested that these cells may have lost their ability to polarize collagen I secretion in the direction of the lens capsule. By P30, picrosirius red labeling is extended to the youngest fiber cells of normal lenses (Fig. 4e, arrow), In contrast, by P30 in N-cad^Δlens^ mice much of the lens tissue now stained for picrosirius red (Fig. 4g, arrow). Immunolabeling of eyes from the P30 N-cad^Δlens^ mouse for collagen I confirmed the excessive production of collagen I by their lenses, and showed that the dysgenic cells of both the lens equatorial epithelium (Fig. 4h, arrow), and remaining cortical fiber cells (Fig. 4h, arrowhead), expressed collagen I.

By immunolabeling sections of wildtype and N-cad^Δlens^ P30 eyes for α-SMA, we next investigated whether lens dysgenesis in postnatal N-cad^Δlens^ eyes also involved the emergence of myofibroblasts, the principle cell type associated with inducing and promoting fibrosis (Fig. 4i–r). α-SMA is a defining feature of the myofibroblasts associated with an aberrant wound-repair response that leads to fibrosis^33,39–41^. No α-SMA-expressing cells were detected in normal P30 lenses (Fig. 4i–m). In contrast, α-SMA-positive cells were a significant population of the dysgenic lenses of N-cad^Δlens^ mouse eyes at P30 (Fig. 4n–r). At this time, α-SMA cells were most highly localized to a region just below the apical surfaces of the lens epithelium in both the anterior (Fig. 4q,r) and equatorial (Fig. 4o,p) zones of the lens, in spaces that had originally been occupied by lens fiber cells. Most of the α-SMA+ cells in these dysgenic lenses exhibited a rounded cell morphology (Fig. 4o,p). However, in a region adjacent to that rich in rounded α-SMA+ cells, located under the anterior lens epithelium, was a population of α-SMA+ cells that had acquired a distinct mesenchymal morphology and contained contractile stress fibers typical of myofibroblasts (Fig. 4q,r).

### Immune cells populate the lens in response to lens degeneration in the N-cad^Δlens^ mouse

The lens has long been viewed as a tissue that is immune privileged^42–45^, similar to other tissue sites like the brain. However, recent work has shown that many tissues generally believed to be inaccessible to immune cells in fact have immune surveillance^46–48^. Since tissue wounding or pathogenesis generally leads to immune cell targeting, and many of the α-SMA+ cells that populate the lenses of N-cad^Δlens^ mice have a rounded morphology typical of immune cells, we investigated if these α-SMA+ cells could be sourced from immune cells. To examine this possibility, sections from the P30 N-cad^Δlens^ eyes were co-immunolabeled for the immune cell adhesion receptor β2 integrin (CD18) and α-SMA (Fig. 4s–x). Most of the rounded α-SMA+ cells were found to co-express β2 integrin+ (Fig. 4s–u, arrows), demonstrating their immune cell origin. However, a subset of the rounded cells that expressed very high levels of α-SMA+ were negative for β2 integrin (Fig. 4s–u, arrowheads). This finding suggested that as the immune cells recruited to these dysgenic lenses continued on their differentiation path to myofibroblasts they begin to lose immune cell-specific molecules. Similarly, α-SMA-stress-fiber-positive myofibroblasts that had acquired a mesenchymal morphology were also negative for β2 integrin (Fig. 4v–x, arrowhead).

**Figure 5:**
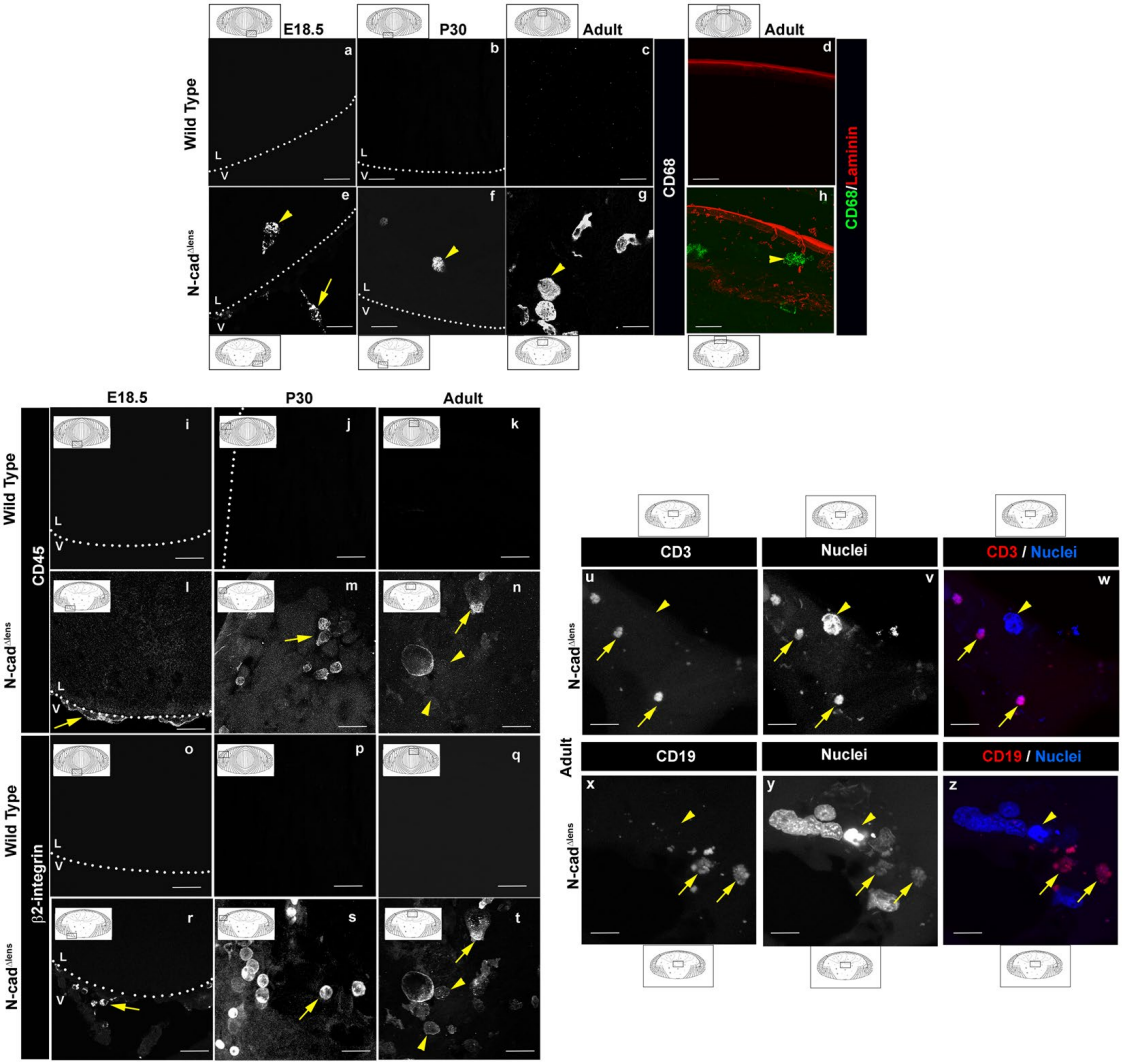
Dysmorphogenesis of lenses from N-cad^Δlens^ mice activates an immune response and invasion of immune cells into the lens. Cryosections of E18.5 (**a,e,i,l,o,r**), P30 (**b,f,j,m,p,s**) and adult (**c,d,g,h,k,n,q,t**–**z**) wildtype (**a**–**d,i**–**k,o**–**q**) and N-cad^Δlens^ (**e**–**h,l**–**n,r**–**z**) eyes were immunostained for CD68 (**a**–**h**), CD45 (**i**–**n**), β2-integrin (**o**–**t**), laminin (**d,h**), CD3 (**u,w**), CD19 (**x,z**) and nuclei (**v,w,y,z**). Lenses from N-cad^Δlens^ eyes showed the presence of CD68-positive cells along the outside of the posterior lens capsule (**e**, arrow) as well as within the lens (**e**, arrowhead), as early as E18.5 that persisted through P30 (**f**, arrowhead) and into the adult (**g,h**, arrowheads), with these immune cells having crossed the laminin-rich lens capsule (**h**). Immune surveillance involving CD45 and β2-integrin-positive immune cells just outside the lens capsule was detected at E18.5 (**l,r**, arrows) with these immune cells invading into the lens by P30 (**m,s**, arrows), that persisted into the adult (**n,t**, arrows). A subset of β2 integrin-labeled immune cells was not labeled for CD45 (**n,t**, arrowhead). Immunolabeling for CD3 (**u,w**) and CD19 (**x,z**), showed that both T cells (**u,v**, arrows) and B cells (**x,z**, arrows) were present in the lenses of adult N-cad^Δlens^ mice. Arrowheads in (**u–z**) denote CD3 or CD19 negative cells. Boxed in regions of the models denote areas represented in the images. (mag bars = 20 μm).

To further examine the link between lens dysmorphogenesis in the N-cad^Δlens^ mouse and the signaling of an adaptive immune response, we conducted a comprehensive analysis of the induction of immune cell surveillance in response to lens dysgenesis. For these studies, sections from E18.5, P30, and adult eyes from both wildtype and N-cad^Δlens^ mice were immunolabeled with immune cell-type specific antibodies, and lenses were imaged by confocal microscopy (Fig. 5a–z). The presence of leukocytes^49–53^ was examined by immunolabeling for both the common leukocyte antigen CD45 and β2-integrin (Fig. 5i–t). The presence of macrophages was investigated by immunolabeling for CD68^54–57^ (Fig. 5a–h), a molecule also expressed by dendritic cells. We found evidence for all three immune cell labels in or around lenses of N-cad^Δlens^ mice as early as E18.5 (Fig. 5e,l,r). At this late stage of lens development in the absence of N-cadherin, CD68 positive cells had already been recruited to areas around the lens (Fig. 5e, arrow) and become located within the dysgenic lens tissue (Fig. 5e, arrowhead). While at E18.5 CD45 and β2-integrin positive cells also were recruited to the region just outside the posterior lens capsule (Fig. 5l,r, arrows), no CD45 or β2-integrin positive cells were detected within the embryonic lens. These results suggest that CD68+ macrophages are the first immune cell responders to lens dysgenesis.

By postnatal day 30 in the N-cad^Δlens^ mouse, CD68+, CD45+, and β2-integrin+ cells were located within the lens (Fig. 5f, arrowhead, m,s, arrows). The increase in recruitment of immune cells to these lenses was consistent with their increasing state of degeneration. Immunolabeling for CD68+, CD45+, and β2-integrin+ in sections from adult N-cad^Δlens^ eyes demonstrated the continued presence of the same complement of immune cells as the lenses continued to deteriorate over time (Fig. 5g arrowhead, n,t, arrows and arrowhead, see Fig. 1f for evidence of state of dysgenesis). Co-immunolabeling for CD68 and laminin, a principle component of the lens capsule, supported the conclusion that these immune cells had populated the lenses of adult N-cad^Δlens^ eyes by crossing the lens capsule (Fig. 5h). Co-immunostaining for CD45 and β2-integrin showed extensive overlap (Fig. 5n,t, arrows), suggesting that a majority of the immune cells recruited to these degenerating lenses were leukocytes. However, there also was a small population of β2-integrin+ cells in these lenses that were not co-labeled for CD45 (Fig. 5n, arrowheads). This result indicated the presence of many distinct populations of immune cells in the adult N-cadherin conditional knockout lens. Immunolabeling of adult N-cad^Δlens^ mouse eyes with antibodies to either CD3 (Fig. 5u,w) or CD19 (Fig. 5x,z) showed that both T cells and B cells had populated these dysgenic lenses. Collectively, these studies suggest that the idea of the lens as an immune privileged tissue must be reevaluated, at least under conditions of dysmorphogenesis, with lens degeneration signaling immune cell recruitment and invasion.

### The lens has access to the lymphatic system

**Figure 6:**
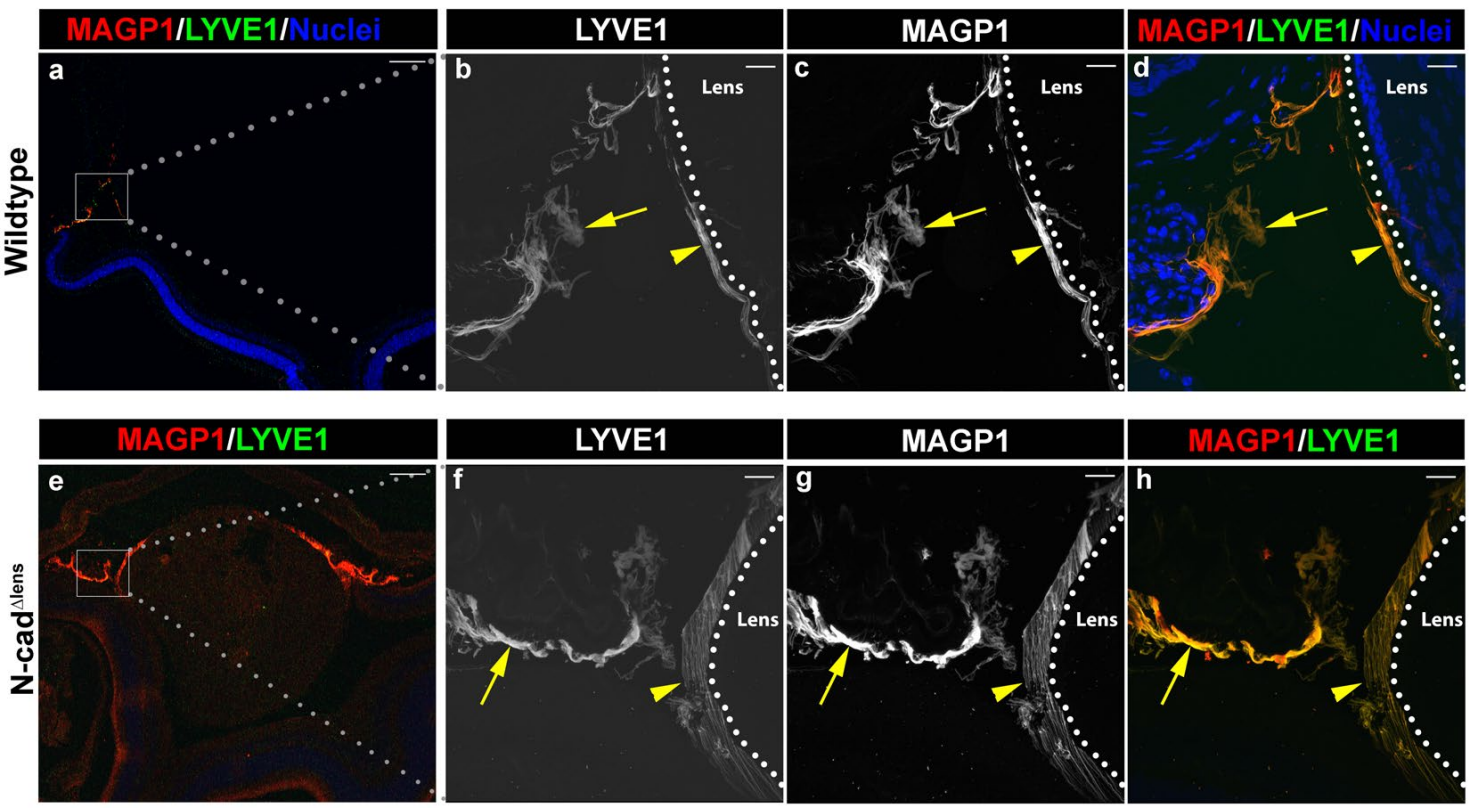
LYVE-1 Positive Labeling Extends along the Lens Zonule Fibers. Cryosections of adult wildtype (**a**–**d**) and N-cad^Δlens^ (**e**–**h**) eyes were co-immunostained for MAGP1 (**a,c,d,e,g,h**), which labels the lens suspensory ligaments, and LYVE-1 (**a,b,d,e,f,h**), a lymphatic endothelial cell surface receptor. LYVE-1-positive labeling was aligned along MAGP1-positive suspensory ligaments in both wildtype (**b**–**d**, arrows) and N-cad^Δlens^ (**f**–**h**, arrows) eyes, and in association with the lens capsule (**b**–**d,f**–**h**, arrowheads). Along the lenses of N-cad^Δlens^ eyes MAGP1-labeled ligaments were thickened (**g**, arrowhead) compared to wildtype controls (**c**, arrowhead), with LYVE-1-positive labeling present throughout this widened ligament structure (**h**, arrowhead). (mag bars in **a**,**e** = 200 μm; in **b**–**d**, **f**–**h** = 20 μm).

Given our remarkable results showing a large immune surveillance and invasion of the dysgenic lens, we wanted to further investigate how these immune cells transit the lens. The lens is a structure known to lack a blood supply^45^, a feature often used to justify its classification as an immune privileged tissue. However, a recent study demonstrated the presence of a lymphatic vasculature in the brain that also extends to the eye^16^. We were therefore curious whether this system extends to the lens. We co-immunostained sections from adult eyes of normal and N-cad^Δlens^ mice using antibodies to lymphatic vessel endothelial hyaluronan receptor 1 (LYVE-1), which is expressed by lymphatic endothelial cells and typically used to detect lymphatic vessels^58,59^, and MAGP1, a microfibrillar protein component of the ciliary zonules, the suspensory ligaments that connect the lens to the ciliary body^60–62^. We discovered that LYVE-1 positive labeling was closely aligned along MAGP1-positive suspensory ligaments in lenses from both wildtype and N-cad^Δlens^ eyes (Fig. 6, arrows in b–d and f–h) and in the ligaments directly associated with the lens (Fig. 6, arrowheads in b–d and f–h). This finding suggested that the lens zonule fibers play a role as supportive structures for the immune system components, and could contribute to the movement of antigen presenting immune cells following lens injury or dysgenesis. Interestingly, labeling of LYVE-1 was more extensive around the lenses of the N-cad^Δlens^ mice than in wildtype lenses, and was correlated with a similar thickening and extension of the MAGP1+ zonule fibers (Fig. 6h, arrowhead). This finding suggests that there may be an increased need for immune cell trafficking in eyes with a degenerated lens.

### CD45/β2-integrin immune cells that populate the dysgenic lenses of adult N-cad^Δlens^ mice continue to be a source of α-SMA+ cells

**Figure 7:**
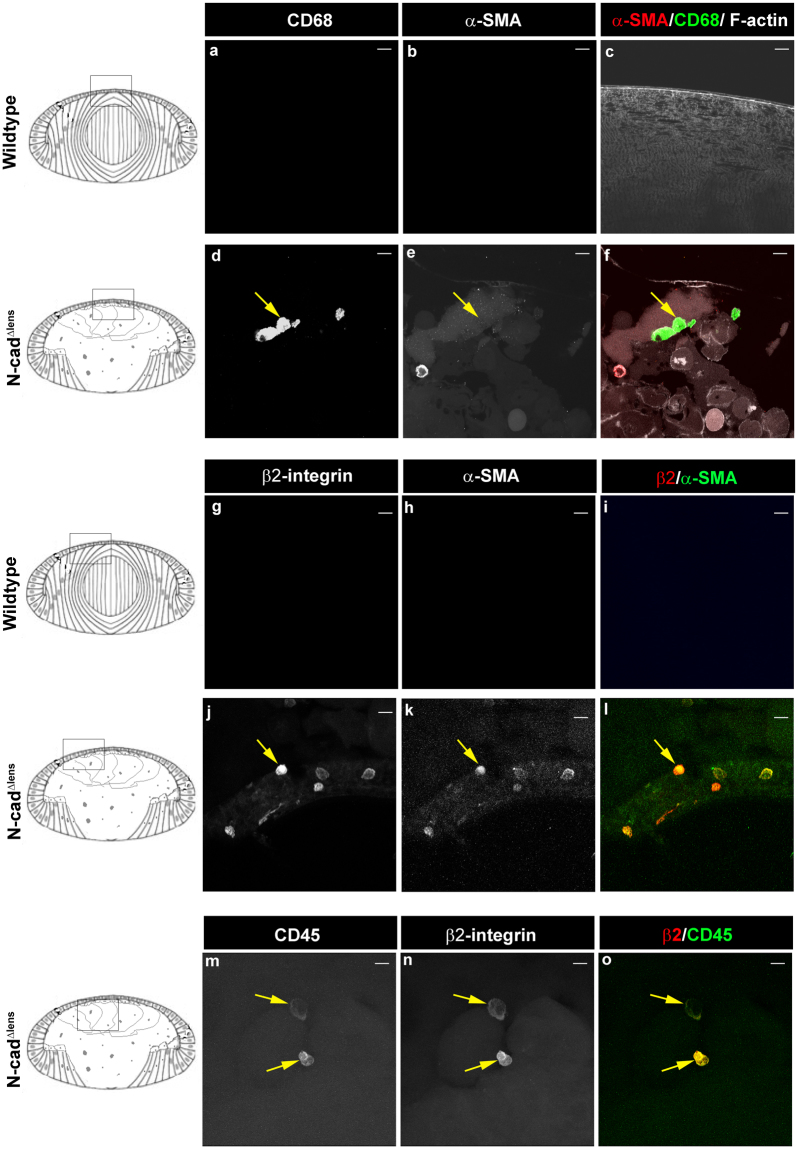
CD45+ Immune Cells are the Principle Source of Myofibroblasts in Lenses of N-cad^Δlens^ mice. Cryosections of adult wildtype (**a**–**c,g**–**i**) and N-cad^Δlens^ (**d**–**f,j**–**o**) eyes were either co-immunostained for α-SMA (**b,c,e,f,h,i,k,l**) with CD68 (**a,c,d,f**) or β2-integrin (**g,i,j,l**), or co-immunolabeled for CD45 and β2 integrin (**m-o**). Sections co-immunostained for α-SMA and CD68 were also labeled for F-actin (**c,f**). Wildtype lenses showed no evidence of immune cell invasion or α-SMA-positive cells. In lenses from N-cad^Δlens^ mice, β2-integrin-positive immune cells were α-SMA positive (**j**–**l**, arrows), but CD68-positive immune cells were not (**d**–**f**, arrows). Co-immunostaining showed a high coincidence of β2-integrin and CD45 co-localization (**m**– **o**, arrows). Boxed in regions of the models denote areas represented in the images. (mag bars = 20 μm).

The interplay between the immune system, inflammation and fibrosis has been well-documented^13,63,64^. Recently, work has highlighted the role of the fibrocyte, a leukocyte subtype that expresses both CD45 and collagen I, in both wound healing and the fibrotic response^65–67^. In the P30 N-cad^Δlens^ mouse, we found that many of the β2 integrin+ immune cells that populated the dysgenic lenses had begun to express α-SMA+, while a subpopulation of cells with a similar rounded morphology that expressed very high levels of α-SMA+ had lost expression of the β2 integrin immune cell receptor (Fig. 4s–u), suggesting that as these cells became myofibroblasts they lost immune molecules. Therefore, we investigated whether immune cells that were recruited to the dysgenic lenses of adult N-cad^Δlens^ mice were also induced to alter their phenotype and express α-SMA, and transition to a myofibroblast phenotype. For these studies, we co-immunolabeled sections of eyes from adult wildtype and N-cad^Δlens^ mice for α-SMA and either CD68 or β2-integrin (Fig. 7a–l). While CD68+ cells in the lenses of adult N-cad^Δlens^ eyes did not express α-SMA (Fig. 7d–f, arrows), most of the α-SMA+ population of these dysgenic lenses were β2-integrin-positive (Fig. 7j–l, arrows). Co-immunolabeling of lenses from N-cad^Δlens^ eyes for CD45 and β2-integrin showed a strong coincident labeling of immune cells in the dysgenic lenses for both these leukocyte molecules (Fig. 7m–o, arrows). The results of these studies suggest that as immune cells continue to be recruited to the dysgenic lenses of the adult N-cad^Δlens^ mouse they are induced to differentiate to myofibroblasts. In addition, it appears that it is a leukocyte population, and not macrophages, that are a primary source of the α-SMA+ myofibroblasts that emerge in response to lens pathogenesis.

### Immune surveillance of the eye in response to lens degeneration

**Figure 8:**
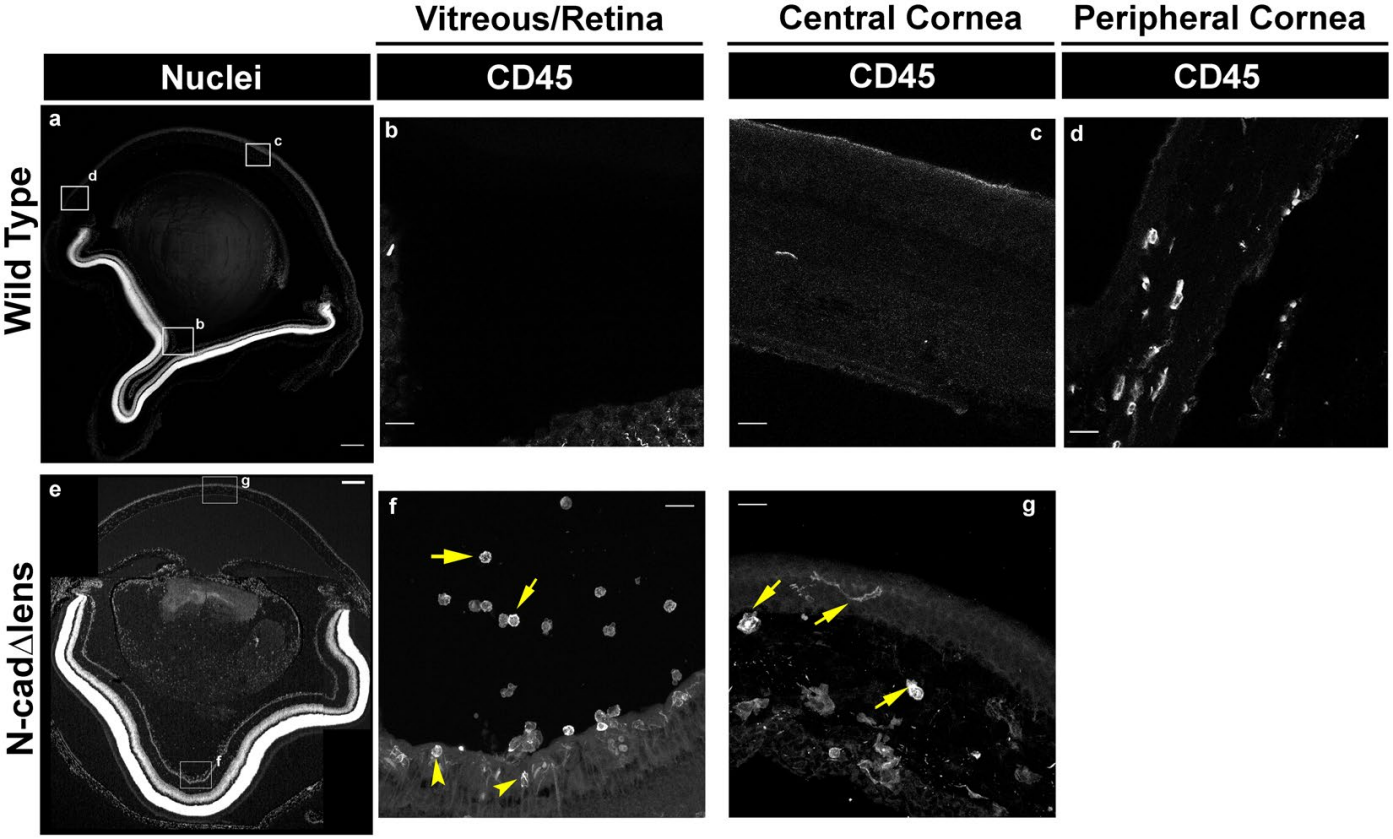
Degeneration of the Lens Activates Immune Surveillance of Other Ocular Tissues. Cryosections of adult wildtype (**a**–**d**) and N-cad^Δlens^ (**e**–**g**) eyes were stained for nuclei (**a,e**) and immunolabeled for CD45 immune cells (**b**–**d**, **f,g**). In the wildtype mouse eye CD45-positive cells were found in the peripheral regions of the cornea (**d**), but no CD45-positive cells were detected in the central cornea (**c**), vitreous (**b**) or retina (**b**). In contrast, in the eyes of N-cad^Δlens^ mice, CD45-positive immune cells were localized to the central cornea (**g**, arrows), vitreous (**f**, arrows) and retina (**f**, arrowheads). (mag bars in **a,e** = 200 μm; in **b,c,d,f,g** = 20 μm).

The visual system relies on integrated function, as well as protection, of its component tissues, the cornea, lens and retina. It therefore interested us to see if the degeneration of the lens resulted in immune surveillance elicited to protect other ocular tissues. To investigate this question, we immunolabeled sections from eyes of adult wildtype and N-cad^Δlens^ mice for the presence of CD45+ immune cells in the cornea, retina, and the vitreous body that is located between the lens and retina (Fig. 8). We observed that the degeneration of the lens in the N-cad^Δlens^ mouse induced immune surveillance of other eye tissues including the central cornea (Fig. 8g, arrows), retina (Fig. 8f, arrowheads) and vitreous humor (Fig. 8f, arrows). This finding demonstrates that lens dysmorphogenesis signals an immune cell response in surrounding ocular tissues, potentially to protect them from degeneration and the eye from further loss of vision. While a somewhat surprising discovery, immune surveillance of the tissues of the visual system in response to lens dysgenesis emphasizes the interplay between these tissues that likely underlies the maintenance of homeostasis in the eye.

## Discussion

N-cadherin has been shown to be critical for lens development^1–4,10^ because of its functions in cell-cell adherence and connectivity, cell migration, and the regulation of cytoskeletal organization. Therefore, tissue-specific dysgenesis resulting from the targeted the loss of N-cadherin provides the opportunity to examine mechanisms related to the failure to maintain tissue architecture that often accompanies both tissue injury and pathological diseases. The N-cad^Δlens^ mouse has provided a unique opportunity to gain this insight since the timing of N-cadherin loss in this conditional knockout allows for lens vesicle formation and the initial process of fiber cell elongation^10^, providing a basic tissue architecture that reacts later to the loss of N-cadherin during secondary fiber cell differentiation. This model allowed us to examine the mechanisms by which a tissue both succeeds and fails in its response to loss of a critical cell-cell junctional protein during development^10^, and now to examine how the extrinsic immune system responds to tissue dysmorphogenesis.

Studies of the loss of other cell-cell junctions in the lens have shown similarities to the effects of loss of N-cadherin in the postnatal mouse, the most general being their development of opacities, highlighting the importance of cell-cell connectivity in the lens^3,31,32,68,69^. Interestingly, the double knockout of Cx50 and Cx46 shows very similar deterioration of the lens as the N-cadherin conditional knockout, with inner fiber cells losing integrity, swelling and degenerating while secondary fiber cells maintain more normal structure^32^. Similarly, loss of Aqp0 also results in fiber cell swelling and loss of cell integrity^31^. The expression of Cx50 and Aqp0 on the membranes of secondary lens fiber cells that maintain connectivity in the N-cad^Δlens^ mouse suggests they may have a coordinated, compensatory function at the lateral cell interfaces of fiber cells in the absence of N-cadherin. Interestingly, although the primary lens fiber cells initially elongate normally in the N-cad^Δlens^ mice^10^, these cells experience the earliest and most severe dysmorphogenesis in the postnatal lens.

Most remarkably, these studies unveil that, contrary to prior beliefs^42,43,70^, the lens is subject to immune surveillance. The presence of LYVE-1 along the suspensory ligaments that connect the ciliary body to the lens in both wildtype and knockout lenses reveals that the lens is connected to the lymphatic system^58,71,72^. Given that the lymphatic system is not only a critical part of the immune system but also integral to tissue homeostasis^73,74^, it is not surprising that the lens, lacking vasculature, would rely on such a mechanism. LYVE-1, the lymphatic vessel hyaluronan receptor expressed by lymphatic endothelial cells, can function as a receptor for hyaluronan, a glycosaminoglycan component of the extracellular matrix^58,59,71^. While hyaluronan has many roles including mediating leukocyte extravasation^58,75^, it has also been suggested to promote posterior capsule opacification^76,77^. The discovery that LYVE-1, an alternative hyaluronan receptor to CD44, is closely associated with the lens could provide insight into hyaluronan’s role in lens injury and wound repair.

Our findings of the presence of immune cells within the lens lead to other insights into lens biology. These immune cells are present within lenses that retain capsular integrity, providing evidence that there must be mechanisms that allow for their movement across the lens capsule. The lens capsule has been thought to contribute to lens immune privilege^78–81^. Our findings that immune cells, highly motile cell populations, first surround the lens and then cross the capsule, provide evidence that the lens capsule is a permeable barrier.

**Figure 9:**
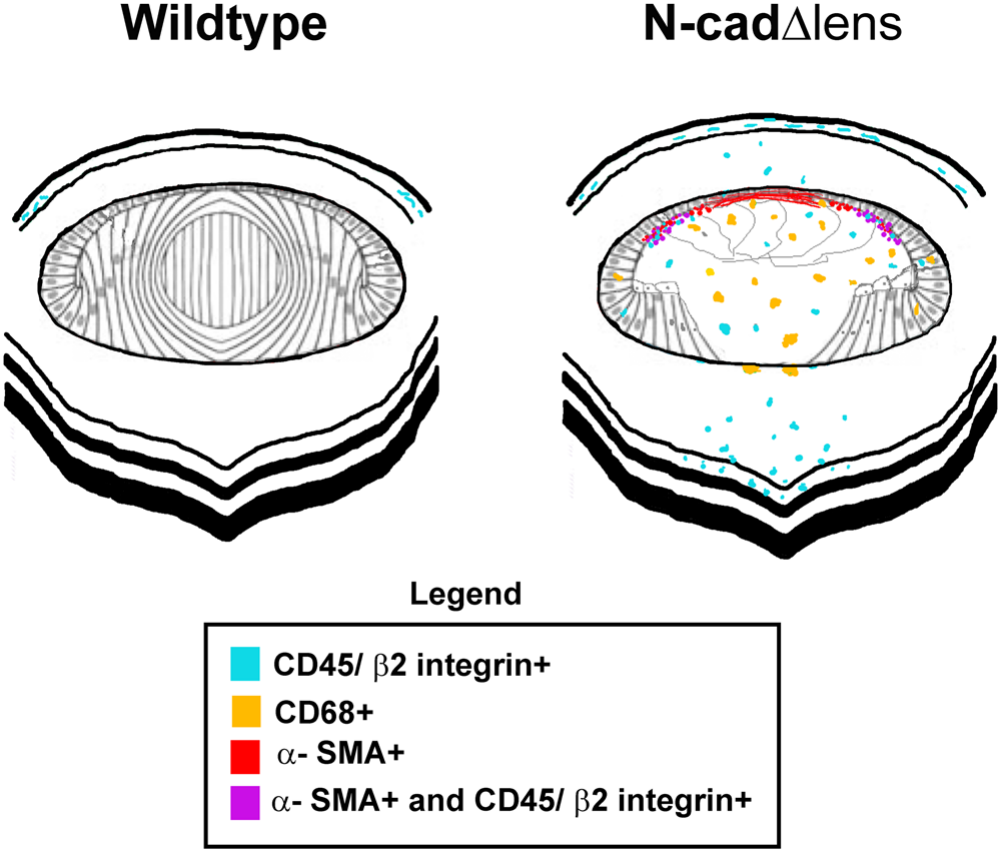
Model of Immune Surveillance of the Dysgenic Lens. In adult wildtype lenses CD45+ cells are present in the peripheral cornea, but not the central cornea. In N-cad^Δlens^ mice the loss of N-cadherin in the lens results in lens degeneration and induces an immune response in lens, cornea and retina. CD45+/β2-integrin+ and CD68+ cells, as well as CD3+ and CD19+ cells, invade into the lens tissue. Immune surveillance of the central cornea, vitreous, and retina is also active. A subpopulation of CD45+/β2-integrin+ cells in the lenses of N-cad^Δlens^ mice with a rounded morphology characteristic of immune cells, located beneath the equatorial epithelium, had begun to express the myofibroblast protein α-SMA. Localizing beneath the anterior lens epithelium, was a population of α-SMA positive cells with a mesenchymal morphology typical of cells that had differentiated to myofibroblasts that appear to have lost the β2-integrin immune cell receptor.

Our studies have only begun to answer the question of the immune system’s role in lens injury response. The presence of CD45, β2-integrin, CD68, CD3, and CD19-expressing immune cells in the lens in response to lens degeneration suggests that this condition elicits a complex immune response (modeled in Fig. 9) and that there are timing differences to how quickly different immune cell types invade the lens, with CD68+ macrophages being the first responders. Another discovery made possible by our studies of the degenerating lenses of the N-cad^Δlens^ mouse is that the α-SMA-positive myofibroblasts that emerge in these lenses were sourced from CD45/β2 integrin-positive immune cells (modeled in Fig. 9) and not the CD68-positive immune cell population. This finding suggests that these different immune cell types have distinct responses to lens tissue pathogenesis. The rapid response of CD68-positive cells suggests that the infiltration of macrophages, which may play roles in phagocytosis, may be recruited to remove dying cells and the extensive proteinaceous material we have found to be produced in the region originally occupied by the primary lens fiber cells, as well as play a role in the recruitment and activation of lymphocytes.

Our finding that the CD45/β2-integrin-positive subpopulation were the principle source of α-SMA myofibroblasts in the degenerating lens of the N-cad^Δlens^ mouse opens the possibility that other fibrotic disorders of the lens associated with cataractogenesis may be the outcome of immune cell recruitment. Interestingly, similar to the lenses of the N-cad^Δlens^ mouse, lenses that develop anterior subcapsular cataracts (ASC) are characterized by fibrotic regions rich in a collagen I matrix and populated by α-SMA+ myofibroblasts located underneath a thickened anterior lens capsule^82^. Since the principal causes of ASC are ocular trauma and inflammation associated with the accumulation of cytokines in the aqueous humor fluid that surrounds the anterior surface of the lens^83^, we speculate that the progenitors of the myofibroblasts associated with ASC could be immune cells recruited to these lenses by injury-induced cytokines.

PCO is a fibrotic condition of the lens that results from an aberrant wound-healing response to cataract surgery. It is characterized by the accumulation of matrix proteins such as collagen I induced by TGF-β^84–86^, and the emergence of α-SMA+ myofibroblasts that we show are the progeny of a vimentin-rich mesenchymal leader cell population recruited to the wound-edge of the cataract surgery-injured lens epithelium^35,87^. In the normal wound response to cataract surgery, these same mesenchymal leader cells direct migration of the epithelium to repopulate the cell-denuded region of lens capsule^35^. Interestingly, the depletion of macrophages prior to cataract surgery prevents the injured lens epithelial cells from populating the posterior lens capsule^88^, providing evidence that the mesenchymal leader cells that differentiate to myofibroblasts could be progeny of immune cells. Since cataract surgery activates an inflammatory response with immune cells populating the aqueous humor, it is likely that these immune cells are recruited to the lens by injury-induced cytokines through the incision made in the anterior lens capsule, and that these cells become PCO-causing myofibroblasts. The link between injury-induced immune cells and myofibroblasts is supported by studies of the fibrotic outcomes to wound-repair in other tissues^13,89^. Inflammatory cytokines like TGFβ are likely an essential element of the recruitment of immune cells to the dysgenic lenses of the N-cad^Δlens^ mouse, a mechanism which we plan to investigate in future studies.

As well as revealing immune surveillance of the lens, our studies of the N-cad^Δlens^ mouse also provided new insight into the interaction and communication that exists between tissues of the ocular system (modeled in Fig. 9). While the N-cadherin conditional knockout is targeted only to the lens, we found that an immune response is elicited in other ocular tissues including the central cornea, vitreous, and retina. These findings suggest that immune surveillance activated throughout the eye following pathogenesis or injury to the lens is a protective response. It stands to reason this organ would have mechanisms that guard its component tissues and prevent an injury affecting one of its components from further compromising the function of the visual system. This finding highlights the importance of investigating processes such as development, injury and repair of a tissue by considering the impact on the larger systems with which it interacts.

## Materials and Methods

### Lens-specific N-cadherin conditional knockout (N-cad^Δlens^) mice

A lens-specific N-cadherin conditional knockout mouse was generated and characterized as previously published^10^. Animal experiments were performed in accordance with the Institutional Animal Care and Use Committee (IACUC) guidelines of Thomas Jefferson University and guidelines of the Association for Research in Vision and Ophthalmology (ARVO) Statement for the Use of Animals in Ophthalmic and Vision Research and the experimental protocol approved by Thomas Jefferson University’s IACUC. For the purposes of these studies, gestational age was determined through detection of a vaginal plug, with day 0.5 (E0.5) of embryogenesis defined as noon of the day of the appearance of the plug. Mice were analyzed at embryonic day (E)18.5, postnatal day (P)30 and as adults, defined as P90 or older. Phenotype of all adult eyes used for these studies was similar. Due to the use of Cre, the observed morphogenetic defects varied in severity; however, the results presented in these studies are representative of the phenotype seen across all N-cadherin conditional knockout mice.

### Immunostaining

Isolated mouse eyes were fixed in 3.7% formaldehyde overnight at 4 °C, cryoprotected in 30% sucrose solution for a minimum of 24 h prior to freezing and 20-μm thick cryosections cut. Sections were incubated in 0.25% Triton X-100 in PBS buffer (2.7 mM KCl, 1.5 mM KH2PO4, 137.9 mM NaCl, 8.1 mM Na2HPO4–7 H2O [Corning, 21-0310CV]) for 12 min, followed by blocking buffer (5% goat or donkey serum, 0.25% Triton X-100) for 1 h prior to labeling. Samples were incubated sequentially in primary antibody diluted in PBS with 0.1% Triton and 3% bovine serum albumin (BSA) at 37 °C overnight, followed by fluorescent-conjugated secondary antibody for 1-2 h at 37 °C (Jackson ImmunoResearch Laboratories, 111-295-144, 115-545-003, 115-295-008), unless primary antibodies were fluorescent-conjugated. Primary antibodies used included: E-cadherin (Cell Signaling, 24E10), βB-crystallin (Santa Cruz, FL-252), connexin 50 (ADI, Cx50-A), aquaporin-0 (ADI, AQP01-A), laminin (Sigma Aldrich, L9393), collagen 1 (ThermoFisher Scientific PA1-26147), α-smooth muscle actin (abcam ab5694), CD68 (BioLegend 103121), CD45 (BioLegend 137011), CD18/β2-integrin (BioLegend 101416), LYVE-1 (eBioscience 53-0443-82), C3 (BioLegend 100212), CD19 (BioLegend 115524), and MAGP1 (Santa Cruz sc-50084). F-actin was localized with Alexa448-conjugated phalloidin (Invitrogen-Molecular Probes). Nuclei were labeled with TO-PRO-3 (Invitrogen-Molecular Probes) and membranes with WGA (LifeSpan BioSciences, LS-C76576).

### Specialized Stainings: TUNEL, Propidium Iodide, Picrosirius Red

*In Situ* Cell Death Detection Kit, TMR Red, Version 11 (Sigma Aldrich) was used for the TUNEL staining assay. Lens cryosections prepared as described above were permeabilized with 0.1% Triton X-100, 0.1% sodium citrate for 2 minutes on ice. Sections were then incubated with TUNEL reaction mixture (45 μl Label Solution+ 5 μl Enzyme Solution) for 1 h at 37 °C according to manufacturer’s instructions. Negative controls were incubated with Label Solution alone.

Lens cryosections were also used for Propidium Iodide (Thermo Fisher Scientific) staining. These lenses were first equilibrated in 2X SSC (0.3 M NaCl, 0.03 M sodium citrate, pH 7.0) followed by incubation in 100 μg/ml DNase-free RNase in 2X SSC for 20 minutes at 37 °C. Following re-equilibration in 2X SSC, lenses were incubated in a 500 nM solution of propidium iodide (stock solution 1:3000) in 2X SSC for 5 minutes. Lenses were then rinsed in 2XSSC and mounted using ProLong© Gold antifade reagent (Thermo Fisher, P36930). Picrosirius Red Stain Kit from Polysciences, Inc. was used for staining collagen I and III in accordance with manufacturer’s protocol.

### Image Analysis

Confocal microscopy was performed using a Zeiss LSM510META or a Zeis LSM800 confocal microscope. Z-stacks were collected, and single optical planes (1.0 µm) selected using the LSM Image Browser or Zen software.
